# Awareness of pubertal body changes among primary school children aged 10–14 years in Eastern Uganda; challenges and opportunities

**DOI:** 10.1186/s12978-022-01466-y

**Published:** 2022-08-19

**Authors:** Sarah Nantono Bunoti, Nazarius Mbona Tumwesigye, Lynn Atuyambe

**Affiliations:** 1 Department of Community Health and Behavioural Studies, Makerere University School of Public Health, Kampala, Uganda; 2 Department of Psychology, Faculty of Education, Kyambogo University, Kampala, Uganda; 3 Department of Epidemiology and Biostatistics, Makerere University School of Public Health, Kampala, Uganda; 4 Department of Community Health and Behavioural Sciences, Makerere University School of Public Health, Kampala, Uganda

**Keywords:** Awareness, Pubertal-related challenges, Opportunities, Pubertal body changes, Primary school children, Eastern Uganda

## Abstract

**Background:**

Globally, programs that educate young people about pubertal body changes are vital. In some communities, teaching sexual education in schools has been the subject of debate. This is probably why access to sexual and reproductive health information and resources is still a challenge to children aged 10–14 years.

**Methods:**

We conducted a qualitative study design among school children aged 10–14 years. Data were collected from 19 focus group discussions (FGDs) in 16 primary schools purposively selected from Eastern Uganda. Data were transcribed, coded and thematically analysed.

**Results:**

We established that girls in rural schools were aware of their body changes than those from urban schools. Boys in urban schools were knowledgeable of pubertal body changes than those from rural schools. We further found that girls experienced pubertal-related challenges amongst themselves and boys including lack of shavers, pain while shaving, rape, bad boy–girl relationships, unwanted early pregnancies, limited funds to buy pads, menstrual pain, etc. Boys too indicated that they experienced similar challenges and these included lack of shavers, pain during and after shaving, changes in height, raping of girls, bad boy–girl relationships, peer pressure, HIV and other STIs, limited infrastructure, voice changes, bad body odour etc. Girls and boys endeavoured to overcome pubertal-related challenges by utilising advise from teachers, parents and friends.

**Conclusion:**

Boys and girls who were knowledgeable about puberty body changes possessed opportunities that enable them to cope with pubertal-related challenges.

## Plain Language summary

Alike, every child is entitled to pubertal-related information irrespective of being-in-school or out-of-school. Children’s understanding of their pubertal body changes is vital in as far as “positive” sexual health is concerned in their adulthood. In this study, we aimed at determining awareness of pubertal body changes, pubertal-related challenges faced by primary school children and opportunities for them to navigate through challenges during puberty. Data was collected from 19 FGDs conducted among boys and girls aged between 10 and 14 years from urban and rural schools in Jinja, Uganda. Our study found that girls in rural schools were aware of their body changes than those from urban schools probably due to the fact that girls in rural schools interact with many close relatives compared to those from urban schools. Pubertal-related challenges experienced by both girls and boys included lack of shavers, pain while shaving, rape, “bad” boy–girl relationships, unwanted early pregnancies, limited financial support, menstrual pain, peer pressure, sexually transmitted diseases, changes in voices, bad body odour etc. Unlike in girls, we found that boys in urban schools were knowledgeable of pubertal body changes than those from rural schools. Over all, our participants navigated through pubertal-related challenges by utilising advise from mainly their teachers, parents and friends. However, boys and girls who were aware of puberty body changes easily navigated through pubertal-related challenges. This therefore means that efforts to create awareness of pubertal-related challenges among young people especially those aged 10–14 years may yield positive results in one’s sexual health during adulthood.

## Background

Globally programs that educate young people about concerns related to pubertal body changes are of great importance. Such programs provide crucial knowledge and skills to help youth navigate the physical, emotional, and interpersonal changes of puberty with positive outcomes [1, 2]. Puberty is a complex, integrative, and coordinated transition, marked by changes in the body, brain, behavior, cognition and emotion [3]. It is a transitional period between childhood and adulthood which includes the process of rapid growth, development and maturation in terms of physical, psychological and social circumstances [4]. It is part of the adolescence, a period of passing through childhood to adulthood [5]. In many parts of the world, children aged 10–14 years are referred to as young adolescents [6]. This age group plays a vital role in the implementation of child-focused programmes [7]. Despite the fact that such programs do increase knowledge among children [1, 8], limited research on awareness of pubertal changes has shown that such knowledge does not seemingly reduce sexual risks among children [8]. Contrary to decades of scientific research on diverse aspects of puberty, awareness of pubertal body changes among children aged 10–14 years has received relatively little attention. In this study we define ‘awareness’ as children’s knowledge of pubertal, physiological and psychological changes during puberty. Some of the changes include breast development, rapid increase in height and weight, growth of pubic hair, widening of hips, menstruation in girls, voice changes in boys, night time ejaculation etc. [9, 10]. In regard to psychological changes, children are uncertain of their emotional state, searching for identity, seeking for independence, starting an intimate relationship are prominent in both girls and boys [1, 10].

In Sub-Saharan Africa (SSA) communities, teaching sexual education in schools has been the subject of debate between abstinence only and comprehensive sexuality education [11, 12]. The debates were mainly centered on three key issues; at what stage to introduce it, what kind of curricula is appropriate and who is qualified to provide such teaching. In East African countries, matters of sex education were traditionally in the hands of grandparents who imparted practical knowledge of sexual practices, family responsibilities, and domestic skills such as the elongation of the labia minora, a cultural experience commonly referred to as visiting the forest/bush [13]. In many Ugandan communities, informal sessions were delivered to girls by older women (often paternal aunts) commonly known as Ssenga, and to boys by uncles commonly known as Koja [14]. Though many developing countries have set up national policies and curricula that support comprehensive sex education in primary schools, there is little data about the extent to which young people actually receive it [1, 15]. That is, little is known about resource allocation to adolescent programming in most developing-country populations [16]. Perhaps this explains why key stakeholders like the UN have not been instrumental in garnering the required participation of children in issues that concern them [17, 18]. Equally, few studies address knowledge of puberty among young adolescents [19] and the content offered may be limited in scope and poor in quality [1, 20].

In Uganda, only 3% and 23% of adolescent females and between 0–13% of adolescent males reported experiences of sexual violence in the past year [15]. This situation has left some children acquire early pregnancies coupled with early parenthood, acquisition of STIs, psychological effects like stress, depression, and trauma among others [18, 21]. Access to sexual and reproductive health information and resources is a challenge to children aged 10–14 years in Uganda [22]. Likewise, many young adolescents are still not receiving such information due to lack of funding and resources to implement programs, cultural controversies around CSE etc. [15, 23]. According to Uganda’s National Sexuality Education Framework, there is still a gap in written materials that outline the appropriate standards for providing sexuality education [24]. Whereas public health campaigns focus on early childhood nutrition and immunization, sexual and reproductive health information is usually reserved for older adolescents [25]. Therefore, in this study we determine awareness of pubertal body changes, challenges and opportunities among primary school children aged 10–14 years in eastern Uganda.

## Methods

### Study design

We conducted a qualitative study among primary school children aged 10–14 years. The design facilitated our understanding of social processes and concepts from the perspectives of study participants, informed by their lived experiences [26–28].

### Population, sample size and site

This study was conducted among children aged 10–14 years attending primary school education in Jinja District. Jinja district is located in the eastern part of Uganda. According to the National Population and Housing Census 2014, the population of Jinja was estimated at about 471,242. Majority of the people in Jinja belong to the Basoga ethnic group; with Lusoga and Luganda being the most widely spoken languages. Jinja District is divided into two administrative units, Jinja Municipal Council (Urban) and Jinja District (Rural). Jinja rural is composed of two counties i.e., Kagoma and Butembe. Primary education in Jinja is under two separate entities; Jinja District Education Office and Jinja Municipal Education Office with a total of 149 primary schools. Sixteen primary schools (ten rural and six urban) were purposively selected to participate in our study. Nineteen FGDs were purposively sampled and each FGD comprised eight participants on average totalling to 152 study participants.

### Data collection

Considering the nature of the study, boys and girls were put in separate groups to enable free expression about puberty. The first author (SBN) conducted 19 focus group discussions (FGDs) comprising ten FGDs for girls and nine FGDs for boys (Table 1). Children were purposively identified from within the selected schools. The FGD guide was pretested to check for accuracy and consistency and to improve validity before actual data collection began. The FGD guide consisted of open-ended questions which elicited responses related to experiences with awareness of pubertal body changes among children aged 10–14 years. Interviews among children were conducted in Lusoga, Luganda and English depending on the preferred language by the participant. Two trained research assistants were recruited to conduct data collection.

**Table 1:** School and focus group discussion characteristics

S/N	Geographical location	Total number of schools	Number of male FGDs	Number of female FGDs	Total number of FGDs	Average number of participants per FGD	Total number of participants
1	Jinja Municipal (Urban)	6	5	5	9	8	72
2	Kagoma County (Rural)	10	4	5	10	8	80
	Total	16	09	10	19		152

### Data analysis

Qualitative data were transcribed verbatim into texts, tallied, coded, grouped (into sub-themes) and tabulated. Tabulated data were categorized into both descriptive and analytical codes using sub-themes developed from the main theme. Tabulated data were later thematically analyzed in line with Creswell’s Six Step of Qualitative Data Analysis [29]. Data matrix (illustrated in Annex 1) was meant to ease data analysis and interpretation processes. Thematic content analysis was desired because it is the most common approach used in qualitative research as it aims at presenting key elements of respondents’ accounts [27]. Findings are presented according to three main themes: awareness of pubertal body changes, pubertal-related challenges and opportunities to navigate through the challenges.

### Ethical considerations

Our study was cleared by the Higher Degrees, Research and Ethics Committee (HDREC) Makerere University, School of Public Health. Thereafter, we also obtained a clearance from Uganda National Council for Science and Technology (UNCST). This study was also cleared by the District Education Office (DEO) Jinja district. SBN presented an introductory letter and a study approval to all the head teachers of the selected schools. Consent was sought from children’s parents/guardians and then an assent was also sought from children themselves before their enrollment in the study. Before SBN began the interviews, she asked participants to read the assent form, she explained the purpose of the study emphasising that participation was voluntary and that they could withdraw from the study at any time. Two copies of an assent form were signed by SBN and the participant; and each participant retained a copy. The FGD guide and assent forms were translated from English to Lusoga for FGD participants who could not comprehend English language. Information about participating schools, respondents’ guardians and the respondents were kept anonymous to ensure confidentiality. We conducted the study in accordance with the four basic research ethics principles of: respect for persons, beneficence (limited harm, sound design and competent investigators), No evil, and justice-morally right, and distributive [30].

## Results

In this section, we report our main findings from three themes including: (a) awareness of pubertal body changes among boys and girls, (b) challenges with pubertal body changes among boys and girls, and (c) opportunities for navigating through the challenges. However much findings from 19 FGDs may not represent all primary school children in Uganda, lessons concerning opportunities to navigate through pubertal-related challenges among primary school children aged 10–14 years may be learnt from this study.

Findings in Table 2 indicated that more Girl’s FGDs (10) were conducted compared to boys’ (09). Then again, more FGDs were conducted in rural schools (10) than in urban schools (09).

**Table 2:** Showing the summary of focus group discussion participants

S/N	Category	Rural	Urban
1	Girls	5	5
2	Boys	5	4
	Total	10	9

### Awareness of pubertal body changes

The main view in this study was that girls from rural schools were aware of the body changes in boys and amongst themselves compared to those from urban schools. On the other hand, girls expressed more body changes in boys than amongst themselves. Unlike girls from urban schools, girls from rural schools revealed that girls are attracted to boys, boys lure girls into sex, boys impregnate girls, boys grow beards, indecent dressing etc.

According to our findings, more girls from rural schools revealed to have experienced pubertal body changes than those from urban schools. Whereas a number of FGDs conducted in rural schools revealed that girls experience attraction to opposite sex, lack of concentration in class, lack of respect for adults and use of herbs to kill the smell, a few girls’ FGDs conducted in urban schools only reported the experience of wet dreams among boys, attraction to opposite sex and lack of concentration in class.

*“Girls starting getting attracted to boys […] they spend a lot of time thinking about boys, even change their walking styles”*, (Girls’ FGD-Rural)

*“I know that girls can get pregnant if they sleep with boys […] girls develop breasts and start having menstruations […]”*, (Girls’ FGD-Rural)

Asked whether they know changes that occur in their bodies as they grow up, boys that participated in our study indicated that they were aware of the body changes among themselves and their counterparts. Unlike their counterparts, boys who participated in five FGDs from urban schools were more aware of body changes in girls and amongst themselves compared to those who participated in four FGDs from rural schools. Generally, many boys from urban areas were aware of the development of deep voice, indecent dressing, wet dreams, and growth of pubic hair/armpit hair among others. However, boys from urban schools were aware of a few body changes of which those from rural areas were not aware of and these included change in voice, change in walking and bad smell. But boys from rural areas were aware of ringworms, human beings who have sex with animals, growth of beards, ability to marry and conceive and indecent dressing and these were not known to boys from urban schools. Irrespective of one’s sex and location of the school, the development of breasts was known to almost all of our study participants.

Generally, most of the boys that participated in our FGDs carried out in urban schools reported to have experienced body changes than boys in rural schools. Boys in urban areas revealed to have experienced changes in their voices, itching of pubic hair, wet dreams, attraction to opposite sex, “big” feeling/feeling that you are in charge, lack of concentration in class, lack of respect for adults and use of herbs to kill the bad smell. Both boys and girls from rural and urban schools concurred that they experience attraction to opposite sex.

*“[…] boys start getting wet dreams […]; wake up in the morning to find shorts wet; but we do not know where the water that wets shorts comes from”* (Boys’ FGD-Urban)

*“I know that girls get attracted to boys and some run away from home to go and get married […] Some girls get hysteria because of their love for boys”,* (Boys’ FGD-Rural).

### Pubertal-related challenges

Most girls from rural schools reported to have faced a number of pubertal-related challenges compared to those from urban schools. Of the five girls’ FGDs conducted in rural schools, pubertal-related challenges faced included: lack of shavers, pain while shaving their private parts, rape, bad boy–girl relationships, unwanted early pregnancies, HIV and other STIs, lack of money to buy pads, painful and irregular menstruation periods, and lack of concentration while in menstruation. A few of the girls that participated in five FGDs that were conducted in urban schools revealed to be faced by rape, bad girl–boy relationships, struggle for boyfriends, love for money, bad body odour, lack of money to buy enough pads, painful and irregular periods and lack of concentration while in periods. Girls from both rural and urban schools who were not in position to buy enough pads reported an alternative of cloth. Girls in rural and urban schools were trying to handle painful menstruation by use of herbs or pain killers.

*“Sugar daddies look at our breasts and start looking at us with enticing eyes; they begin disturbing us with unwanted pregnancies […]”*, (Girls’ FGD-Rural)

*“[…] I do not pay attention in class due to pain just before my periods or during my periods; people can know that I am in periods […]”,* (Girls’ FGD-Rural)

*“We sometimes resort to using pieces of cloth […] we do not use pads because we cannot afford to buy them all the time […]”*, (Girls’ FGD-Rural)

*“We have boyfriends and we have sex, they give us money and we eat it […] some girls in my class have been fighting for such boys”*, (Girls’ FGD-Urban)

Pubertal-related challenges that were faced by boys in urban schools were more compared to those faced by boys in rural schools. Boys in five FGDs that were conducted in urban schools revealed a number of challenges including lack of shavers, feeling pain while shaving, lack of saloons for pubic hair, change in height, raping girls, bad boy–girl relationships, peer pressure, unwanted pregnancies, HIV/STIs, limited infrastructure, voice changes, and bad body odour. A few boys in one urban school wondered why there are no saloons for shaving private parts. The general consensus was that they would instead visit such saloons. Boys that participated in four FGDs which were conducted in rural schools reported to be faced with pain while shaving their private parts, impregnating young girls, HIV/STIs, casual work, and changes in their voices. Because of the pain they go through, a number of boys seemed to have shunned the habit of shaving their private parts. Boys from only rural schools revealed that they engage in casual work for purposes of earning money to manage pubertal changes. Boys work as casual labourers at construction sites, carry luggage in markets, work in sugar cane plantations etc. Largely, boys from both rural and urban schools reported that their feelings for girls force them to initiate love relationships with them. Boys tell girls how they love them especially when they are called to give a hand e.g. solving a mathematical problem. However, boys reported that they fear to make them pregnant. In general, boys from both rural and urban schools were not comfortable with the changes in their voices.

*“I have arm pit hair […] they make me sweat; It also causes a bad smell” […] I fear sitting close to other people because they will not like the smell”*, (Boys’ FGD-Rural).

*“We also have a problem of buying blades for shaving; in most cases we have no money for buying them but teachers insist that we have to shave […]”*, (Boys’ FGD-Rural).

*“But even when we buy razor blades, it is very painful. Sometimes I cut myself accidentally when shaving and I believe it is a problem because it is not easy to see your private parts when shaving […]”*, (Boys’ FGD-Rural).

*The girls ask for help in drawing maps for them, help with mathematics […] as we help, we get attracted to them and we cannot avoid falling in love”*, (Boys’ FGD-Rural).

### Opportunities for navigating through pubertal-related challenges

#### Pubertal information and teacher–child communication

Boys and girls navigated through pubertal-related challenges with the help of readily available pubertal information. Asked about how they got to know their body changes, girls from both rural and urban schools revealed that they were getting pubertal-related information from their teachers. Senior man/woman teachers availed pubertal information through their advice to pupils on how to manage their body changes. Unlike girls in urban schools, girls in rural schools revealed that their teachers advised them on their personal hygiene. Besides girls from one FGD conducted in a rural school reported that their teachers advised them on how to behave well while managing their body changes. Teachers were perceived to be supportive in regard to enriching children with pubertal-related information. Girls from both rural and urban schools further reported that they received pubertal information from fellow girls.

*“The boys and girls are put in separate groups […] girls are taught by the senior women teachers and the boys are taught by senior men teachers […]”*, (Girls’ FGD-Rural).

*“I saw a girl whose school uniform had blood spots […] I asked other girls and I was told that she was in her menstruation periods”*, (Girls’ FGD-Urban).

Boys in urban schools were availed with pubertal-related information more than those in rural schools. Boys from four FGDs (urban) and one FGD (rural) revealed that they get opportunities to know their body changes through their teachers’ advise. Findings from three FGDs (urban) and one FGD (rural) indicated that boys are also advised by teachers on how to manage their body changes. Boys and girls in both rural and urban schools confirmed that they have an opportunity to ask their teachers questions related to body changes. Teachers were believed to be performing a good job of enlightening pupils about their personal hygiene as illustrated by the following extract:

*“Teachers have advised us to use shaving sticks and not mere razor blades in order to avoid body itching […]”*, (Boys’ FGD-Urban).

#### Parent–Child communication

Findings from our study revealed that girls from both rural and urban schools were receiving pubertal-related advice from their parents and other close relatives. On the other hand, other girls perceived their parents to be uncooperative, unapproachable when it comes to communicating about body changes.

*“I talk to my mother and my older sister because I know that they have gone through the same experiences […]”*, (Girls’ FGD-Urban)

A good number of boys from three FGDs (urban) and one FGD (rural) indicated that they have an opportunity of communicating with their parents. They are advised by their parents and close relatives. Although boys and girls from both rural and urban schools believed that their parents were not sharing information about body changes, not willing to talk about body changes, the general consensus was on parental advice.

*“[…] my parents tell me about the changes in behavior and warn me never to behave in the same way, even my grandmother talked to me […]”*, (Boys’ FGD-Urban).

When asked about other opportunities of getting pubertal related information, both boys and girls from rural and urban schools indicated friends as the best other source of information compared to Radios, Televisions, newspapers (Straight talk), Health workers, Sunday school etc.;

*“We talk about body changes with friends; we observe changes in boys and girls […]; I have seen my pubic hair […], my voice has changed too”*, (Boys’ FGD-Urban).

*“We got to know about these changes by observing the way other boys look and behave; we also talk about these changes with our friends in our groups”*, (Boys’ FGD-Rural).

#### School support system

Findings from six girls’ FGDs conducted in both rural and urban schools confirmed support extended to children by their senior man/woman teachers. Few girls from urban schools reported to have received support from non-governmental organisations (NGOs) such as Straight Talk Foundation.

Boys from both rural and urban schools appreciated support given to them by NGOs. Boys from an urban school only reported support from school clubs and churches. Children in schools utilize school clubs to share information about their body changes. A well-established school support system to manage body changes among children is believed to be equipped with pads, spare uniform in case of blood stain, pain killers, information on body changes etc.

#### School protection mechanisms

Girls from rural schools reported a number of protection mechanisms compared to those from urban schools who were not in position to cite any. Girls who participated in one FGD (rural school) recognized the role of prefects. When a pupil has pubertal-related challenges, a prefect is in position to report such cases to teachers for action. Other girls from another FGD conducted in a rural school still revealed that there is limited interaction between boys and girls. A few girls from urban schools indicated that school rules and regulations act as a measure to tame pupils’ behaviour while managing their body changes.

*“Some prefects act like office massagers to administration; they deliver our issues to administration […], sometimes, we are helped by these prefects”*, (Girls’ FGD-Rural)

*“The school rules do not allow us to couple with boys […] if found with men outside the school gate, you are automatically disqualified […]”*, (Girls’ FGD-Urban)

According to the boys who participated in our FGDs from both rural and urban schools, keeping children busy with chores is a great occasion when it comes to managing body changes. A few of the boys from an urban school reported the availability of separated bathrooms for big and small boys. Boys in one of the FGDs conducted in a rural school only revealed that school prefects help them to report some of their pubertal related challenges to teachers. Still, boys from one FGD conducted in a rural school reported that girls and boys do not share rooms (such as bathrooms, resting rooms etc.).

#### Knowledge of children’s rights and responsibilities

Both boys and girls in only urban schools demonstrated to be knowledgeable about their rights and responsibilities. Majority of the boys reported to be knowing their right to education followed by their right to health.

*“[…], by the way, I know my parents are supposed to educate me until I grow up; it is my right to go school irrespective of my background”*, (Boys’ FGD-Urban).

Asked about their sources of knowledge on children rights, both boys and girls from only urban schools looked at their teachers as their best source. The other sources of knowledge on rights reported were fellow children and parents.

*“Our teachers have been telling us about these things of rights for us to be educated, to be given good healthcare […]”*, (Girls’ FGD-Urban)

## Discussion

This study explored primary school children’s awareness of pubertal body changes, pubertal-related challenges faced, and opportunities to navigate through the challenges. Evidently, this study showed that girls from rural schools were knowledgeable about body changes than those from urban schools. This has probably rendered girls from rural schools to experience a number of body changes than those from urban schools. This can be partly explained by family settings in rural areas where girls may have an opportunity of living with a number of relatives that might provide pubertal-related information. Our findings are contrary to Jain and a colleague who found increased awareness of pubertal changes among urban adolescent girls than in rural adolescent girls [31]. On the other hand, boys from urban schools were more aware of the body changes compared to those from rural schools. Similarly, most boys in urban schools reported to have experienced body changes than those in rural schools. This is an indication that boys from urban schools receive more pubertal-related information than those from rural schools. Nevertheless, findings of this study demonstrated that almost all study participants were aware of the development of breasts in girls. In line with a study conducted among primary school children, most reported physical body changes included growth of breasts [32]. Largely, our study participants concurred that they experienced attraction to opposite sexes confirming that it is during the age range of 10–14 years when young people are eager to learn the experiences of the opposite sex [33, 34], and the time when they experience significant body changes [35]. Similarly, Uganda’s National Sexuality Education Framework points out that, ages 10–14 years is the time for understanding how the body feels before the on-set of menstruation, preparing appropriately for one’s monthly menstrual periods, maintaining personal and environmental hygiene and knowing signs of poor menstrual hygiene [24].

Our study found that girls from rural schools faced more pubertal-related challenges than those from urban areas. Expectedly, children from rural areas are believed to lack essential resources to prevent some avoidable pubertal-related challenges. Girls studying in urban schools are mostly from privileged families compared to their counterparts. For instance, girls from rural schools who were not in position to buy sanitary pads opted for cloth. A similar study on menstrual management in Uganda showed use of old cloth as means of coping among girls [36]. Our findings support president Yoweri Museveni’s pledge to supply free sanitary pads to primary school girls in an effort to curb down absenteeism [37]. In terms of reported challenges, non-equal distribution of resources ought to be considered while allocating resources to rural and urban schools. This means that more attention ought to be paid to girls in rural schools. However, girls in both rural and urban schools reported to have utilised herbs and sometimes pain killers to avert menstrual pain. Likewise, menstrual pain was a key concern of girls as it was affected by their limited access to analgesics and the widespread belief that the use of analgesics would be detrimental to their health [38]. On the other hand, boys in urban schools faced more pubertal-related challenges than those in rural schools. Among the challenges raised was pain while shaving. In regard to pain, some boys seemed to have shunned the practice of shaving. It is implied that boys compromised their personal hygiene at the expense of limited knowledge and resources. This is probably why boys engaged in casual work to earn money to buy, for instance, good shavers that are believed to prevent pain and scratchy feelings during and after shaving. Nonetheless, boys need advice about how to shave and when to start shaving [39].

Availing boys and girls with information about their body changes is among the opportunities to overcome pubertal-related challenges [40]. Our study participants received pubertal-related information from senior man/woman teachers, parents and fellow children. Senior man/woman teachers availed pubertal information through their advice on how to behave well while managing body changes. Teachers also provided children with information about their rights such as right to education, health etc. Likewise, the Ugandan government has suggested that senior women teachers as among the ways to improve gender discrimination which helps girls overcome numerous challenges [41]. Contrary to our findings, other studies found out that mass media, peers, class lessons and school seminars were the common sources of information about body changes [34, 42]. Studied children confirmed opportunities of asking their teachers questions about body changes. Besides teachers, children received pubertal-related information from their parents and close relatives. Our findings demonstrated that some parents have taken keen interest in talking about body changes with their children. Based on these findings, we can therefore affirm that there is some level of communication between parents and their children about body changes [42]. Furthermore, friends were reported as the best other source of pubertal-related information. Consistent with our findings, some studies conducted in Nigeria revealed similar sources of pubertal-related information to include friends/peers among others [43, 44]. There is a need for a well-established school health program to manage body changes among children with the main focus on sanitary pads, spare school uniform, pain killers, information on body changes etc. Central to this argument, gaps in school health programs in Uganda have constrained the education system with limited access to SRH information and services for young people [20]. More so, girls reported a number of protection mechanisms including the role of prefects who reported pubertal-related challenges to teachers. These findings mean that prefects were playing a role of bridging the information gap between children and teachers. Also, it should be noted that being knowledgeable about your rights and responsibilities is one’s great opportunity to have control over your physical health [40] to develop a positive body image [35]. Generally, school children’s knowledge about their right to health, education etc. puts them in a position to demand pubertal-related information from school administrators, parents, relatives, friends etc.

### Policy implications

As the saying goes: ‘*knowledge is wealth*’, teachers, parents and children need to be sensitized and appreciate the fact that when a child is knowledgeable about certain body changes, he/she might cope well with challenges that come along with these body changes. For instance, sensitized girls take menstrual pain to be normal as they try to avoid use of herbs and some pain killers that may in turn bring about complications that are expensive to treat.

Boys and girls especially those aged 10–14 years should endeavor to acquire knowledge about body changes amongst themselves in an effort to prevent avoidable pubertal-related challenges. Like this study found, children need constant encouragements to ask their senior man/woman teachers, parents, close relatives, friends etc. questions related to their body changes.

Our findings showed an opportunity of sharing information on body changes between teachers, parents and children. It is evident that parents have seen the importance of puberty-related discussions with their children. Parents ought to encourage their children to ask them questions related to body changes. By this green light, children might be in position to open up.

## Conclusion

This study explored awareness of pubertal body changes, pubertal-related challenges and opportunities among primary school children aged 10–14 years in Eastern Uganda. Findings of this study demonstrated that boys and girls who were aware of puberty body changes navigated through pubertal-related challenges by utilizing a number of opportunities such as taking senior man/woman teachers’, parents’ and friends’ advice.

## Data Availability

The datasets used and/or analysed during the current study are available from the corresponding author on reasonable request.

## Annex 1. Data matrix for boys’ and girls’ FGDs

**Table:**

Broad themes	Descriptive and analytical codes	Boys’ responses									Girls’ responses										Total response	Total FGDs
		Rural school				Urban school					Rural school					Urban school						
		1	2	3	5	1	2	3	4	5	1	2	3	4	5	1	2	3	4	5		
Awareness																						
Awareness of pubertal body changes in boys	Deep voice	0	0	9	2	3	10	6	12	13	0	0	0	0	5	7	0	0	0	0	67	19
	Pimples in boys and girls	2	0	4	0	0	5	0	0	0	0	0	8	0	5	7	0	7	0	0	38	19
	Ringworms	6	0	0	0	0	0	0	0	0	0	0	0	0	0	0	0	0	0	0	6	19
	Muscular/authority/penis enlarges	5	5	3	0	0	9	0	0	0	0	0	0	8	0	7	0	0	0	7	44	19
	Attraction to opposite sex	4	0	0	0	0	6	0	8	0	0	0	8	7	5	0	0	0	0	0	38	19
	Defile and are also defiled	3	0	0	2	2	0	0	0	0	0	0	8	6	0	7	0	0	0	0	28	19
	Lure girls to sex	4	0	0	8	1	5	0	8	0	6	0	0	0	0	0	0	0	0	0	32	19
	Go dancing	5	6	0	0	1	0	0	0	0	0	0	0	0	0	0	0	0	0	0	12	19
	Impregnate girls	6	0	0	0	1	0	0	0	0	0	0	8	0	0	0	0	0	0	0	15	19
	boys/girls walk at night and exposed to abuse	6	0	0	0	1	7	0	0	0	7	0	8	0	6	7	0	0	0	0	42	19
	Drink	6	0	0	0	5	0	0	0	0	0	0	0	0	0	0	0	0	0	0	11	19
	Sex with animals	6	0	0	3	0	0	0	0	0	0	0	0	0	0	0	0	0	0	0	9	19
Awareness of pubertal body changes in girls	Pubic hair	0	0	2	0	5	7	6	0	10	6	7	0	8	0	0	7	7	0	7	72	19
	Active in love relationships	6	5	2	4	5	0	0	5	0	6	8	8	8	0	7	0	0	0	0	64	19
	Develop breasts	0	7	5	0	3	4	6	6	8	0	7	14	8	6	7	7	7	7	0	102	19
	Change in voice/walking	0	0	0	0	2	0	6	0	0	0	0	0	8	6	7	7	0	0	0	36	19
	No respect for adults, teachers	0	5	6	0	3	0	0	6	0	0	0	0	0	0	0	0	0	0	0	20	19
	Able to conceive and marriage	0	5	0	0	0	0	0	0	0	0	0	0	0	0	0	0	0	0	0	5	19
	Bad smell	0	0	0	0	0	5	0	0	0	0	0	8	0	7	7	7	0	0	0	34	19

Rural schools: (1) Budondo C/U Primary School; (2) Busiya Parent's Primary School; (3) Kivubuka Primary School; (4) Lukolo Muslim Primary School; (5) St. Mary’s Nsuube Primary School

Urban schools: (1) Jinja Army Day and Boarding Primary School; (2) Jinja SDA; (3) Kiira Primary school; (4) Spire Road Primary School; (5) Victoria Nile Primary School

## Abbreviations

FGDs: Focus group discussions
SRH: Sexual reproductive health
WHO: World Health Organization
CSE: Comprehensive sexual education
